# Advances in genetic profile and therapeutic strategy of pulmonary large cell neuroendocrine carcinoma

**DOI:** 10.3389/fmed.2024.1326426

**Published:** 2024-02-27

**Authors:** Siyu Zhu, Xinyue Wang, Hui Li, Peiyan Zhao, Jingjing Liu, Liang Zhang, Ying Cheng

**Affiliations:** ^1^School of Traditional Chinese Medicine, Changchun University of Traditional Chinese Medicine, Changchun, Jilin, China; ^2^Postdoctoral Research Workstation, Jilin Cancer Hospital, Changchun, Jilin, China; ^3^Translational Cancer Research Lab, Jilin Cancer Hospital, Changchun, Jilin, China; ^4^Jilin Provincial Key Laboratory of Molecular Diagnostics for Lung Cancer, Jilin Cancer Hospital, Changchun, Jilin, China; ^5^Department of Thoracic Oncology, Jilin Cancer Hospital, Changchun, Jilin, China

**Keywords:** pulmonary large cell neuroendocrine carcinoma, molecular subtype, biomarkers, therapeutic advances, immunotherapy

## Abstract

Pulmonary large cell neuroendocrine carcinoma (LCNEC) is a high-grade neuroendocrine carcinoma (HGNEC) accounting for 3% of primary lung cancer, and characterized by strong invasion, high heterogeneity, and extremely poor prognosis. At present, the diagnosis and treatment of LCNEC remains controversial and refer to therapeutic strategy of small cell lung cancer (SCLC), lacking precise therapy. Recently, the genetic analysis and clinical trials of LCNEC gradually emerged, providing more evidence for precise diagnosis and treatment. Here, we review the diagnosis, molecular characteristics, and treatment of LCNEC based on the existing research and frontier progress to provide a potential direction for future diagnosis and treatment of LCNEC.

## 1 Introduction

Pulmonary large cell neuroendocrine carcinoma (LCNEC), a rare pathological type of lung cancer, with high invasiveness and poor prognosis, classified into high-grade neuroendocrine carcinomas (HGNEC). Although LCNEC merely accounts for 2.4−3.1% of lung cancer, only 25% of the patients are early stage, while 40−50% are advanced metastatic stage when diagnosis. The median overall survival (mOS) of LCNEC patients are 36 °months, and the 3-year and 5-year survival rates are 49 and 44.7%, respectively, (1). The majority of LCNEC patients are male (about 62.5%), with an average age of about 65°years old and a history of heavy smoking (2). LCNEC was once categorized as a variant of large cell carcinoma, with the increased understanding, it was classified as neuroendocrine carcinoma in 2015 by World Health Organization (WHO). The diagnostic criteria of LCNEC are mainly based on cell morphology and neuroendocrine differentiation. In detail, large cells, low nuclear/plasmic ratio, vacuolated chromatin with obvious nucleoli, organoid, fenestrated and rosette structures, as well as the identification of neuroendocrine morphology and the expression of at least one of the neuroendocrine markers (chromogranin A, synaptophysin, or CD56) or show endocrine characteristics under the electron microscope, are criteria for the diagnosis of LCNEC. However, diagnosis by morphology through neuroendocrine markers is not able to differ LCNEC from other lung neuroendocrine carcinomas, such as small cell lung cancer (SCLC), increasing the risk of underdiagnosis and misdiagnosis of LCNEC. Additionally, the low prevalence rate and small sample size have resulted in finite research on genotype and biomarkers for LCNEC.

Due to the limited understanding and low incidence of LCNEC, there is currently no standard treatment guideline from prospective clinical trials. Surgery is recommended for early-stage LCNEC patients (TNM I-III, AJCC 8th edition), while chemotherapy for patients with medium-term and advanced stage. The chemotherapy regimens include etoposide-cisplatin (EC) and etoposide-platinum (EP), or platinum-gemcitabine/paclitaxel (GEM/TAX) and gemcitabine/paclitaxel regimens, the former is used in SCLC and the latter used in non-small cell lung cancer (NSCLC). However, the high heterogeneity of LCNEC has resulted in contradictory and limited efficacy of therapeutic regimens across different clinical trials (3). Therefore, it is imperative to seek more effective treatment options and potential therapeutic targets and improve prognosis of patients with LCNEC. This review is to provide an overview on the genetic profile and clinical advances of LCNEC to enhance the understanding of its biology, prognostic implications and therapeutic strategies.

## 2 Diagnosis of LCNEC

Large cell neuroendocrine carcinoma (LCNEC) was included in HGNEC, which proposed by WHO and the International Association for the Study of Lung Cancer (IASLC) (4, 5) (Table 1), with a neuroendocrine morphology including lobules, organoid or trabecular patterns, rosette-like structures, or peripheral palisading, accompanied by extensive necrosis and a high proliferation rate. Accurate diagnosis of LCNEC requires large surgical specimens and the use of morphological and endocrine markers. However, the histological features of LCNEC overlapped with NSCLC and SCLC, increasing the difficulty of histological diagnosis (6, 7). The result of a phase II clinical trial showed that approximately 27.5% of the subjects initially diagnosed with LCNEC were reclassified as other types of lung cancer, predominantly SCLC. In another study, only 53% (*n* = 44) of patients were initially correctly classified as LCNEC, while 47% (*n* = 37) were misdiagnosed as NSCLC (8, 9). Therefore, LCNEC needs to be distinguished from NSCLC and SCLC by clinicopathological and molecular features (Table 2).

**TABLE 1:** Grading Systems for NENs of the lung and thymus adapted from IASLC 2018 and WHO 2022.

Neuroendocrine neoplasm	Classification	Grade	Diagnostic criteria		
			**Mitotic counts per 2°mm^2^**	**Necrosis**	**Ki67 index**
Well-differentiated (NET)	Typical carcinoid/NET grade 1	Low	>2	No necrosis	−
	Atypical carcinoid /NET grade 2	Intermediate	2−10	And/or necrosis (usually punctate)	−
	Carcinoid/NETs with elevated mitotic counts and/or Ki67 proliferation index	-	>10	-	>30%
Poorly-differentiated (NEC)	Small cell (lung) carcinoma	High	>10	Often necrosis and small cell cytomorphology	−
	Large cell NEC	High	>10	Virtually always necrosis and large cell cytomorphology	−

NEN, neuroendocrine neoplasm; WHO, World Health Organization; IASCL, International association for the study of lung cancer; NET, neuroendocrine tumor; NEC, neuroendocrine tumor carcinoma.

**TABLE 2:** Comparison of clinicopathological and molecular features of LCNEC, SCLC, and NSCLC.

	LCNEC	SCLC	NSCLC
% of lung tumors	2−3%	13−17%	76%
Histopathological features	Cell size 3 × lymphocytes diameter Abundant cytoplasm Prominent nucleoli Frequent necrosis	Dense proliferation of small tumor cells, scant cytoplasm, finely granular chromatin, inconspicuous nucleoli, nuclear molding, extensive necrosis, crushing artifacts	LUAD: glandular differentiation or mucin production LSCC: squamous cell differentiation (i.e., keratinization, keratin pearl formation and intercellular bridges) with moderate to abundant cytoplasm
Biomarkers	Neuroendocrine markers CgA, NCAM/CD56, Syn (80−90%) TTF-1 (50%)	Neuroendocrine markers CgA, NCAM/CD56, Syn (80−90%) TTF-1 (85%)	LUAD: TTF-1 (>85%), NapsinA LSCC: p40, CK5/6 (p63)
Molecular patterns	Type I (*TP53, KEAP1, STK11*) Type II (*TP53* and *RB1* co-inactivation)	SCIC-A (*ASCL1*) SCLC-N (*NEUROD1*) SCLC-P (*POU2F3*) SCLC-Y/I (*YAP1/*Inflamed)	Six molecular subtypes in LUAD (83), four in LSCC (84)
Standard first-line	No standard regimen, SCLC chemotherapy and NSCLC chemotherapy	Platinum plus etoposide	LUAD (Platinum plus pemetrexed) LSCC (Platinum plus gemcitabine/paclitaxel) TKI in oncogene-addicted
Five-year survival rate	Early stage (21−62%) (85) Advanced stage (10%) (86) Overall (15−57%) (85)	LS-SCLC (10−13%) (87) ES-SCLC (1−2%) (87) Overall (6.7%) (88)	Early stage (<87%) (89) Limited stage (15−32%) (87) Advanced stage (<20%) (90) Overall (26.3%) (88)

LCNEC, large cell neuroendocrine carcinoma; SCLC, small cell lung cancer; NSCLC, non-small-cell lung cancer; CgA, Chromogranin A; NCAM, neural cell adhesion molecule; Syn, Synaptophisin; TTF-1, thyroid transcription factor-1; CK5/6, Cytokeratin 5/6; LUAD, lung adenocarcinoma; LSCC, lung squamous cell carcinoma; *TP53*, tumor protein p53; *RB1*, Retinoblastoma gene-1; *KEAP1*, kelch-like ECH associated protein 1; *STK11*, serine/threonine kinase 11; *ASCL1*, Achaete-scute homolog 1; *NEUROD1*, neurogenic differentiation factor 1; *POU2F3*, POU class 2 homeobox 3; *YAP1*, yes-associated protein 1; TKI, tyrosine-kinase inhibitor; LS-SCLC, limited stage-small cell lung cancer; ES-SCLC, extensive stage-small cell lung cancer.

Reliable diagnostic biomarkers are essential for LCNEC, which typically shows high expression of multiple neuroendocrine markers, including neural cell adhesion molecule (NCAM/CD56), synaptophysin (Syn), chromogranin A (CgA), etc. Among them, CD56, expressed in 92−98% of LCNEC, may be the most sensitive marker for the diagnosis of LCNEC, but also expressed in nearly 10% of adenocarcinoma, squamous cell carcinoma, and large cell carcinoma, leading to low specificity. In contrast, CgA is expressed in nearly 70% of LCNEC and may be the most specific marker, lacking sensitivity conversely. Syn is expressed in 87% of LCNEC, nevertheless, in 10% of adenocarcinomas and 5% of squamous cell carcinomas (10). The combination of the three traditional neuroendocrine markers increased the sensitivity for LCNEC from 47 to 93% on paired biopsy specimens (11). Additionally, other potential diagnostic markers have been investigated. Insulinoma-associated protein 1 (INSM1) is a nuclear transcription factor, expressed in neuroendocrine neoplasms (NENs). Multiple studies had shown that INSM1 was expressed in 68−91.3% of LCNEC (12–14). The sensitivity of INSM1 (75%) in LCNEC was higher than that of CgA (46%), and the specificity (97%) was similar to that of CgA (98%), but higher than that of Syn (90%), and CD56 (87%) (15). Möller et al. (16) found that INSM1 was used as an additional marker, the sensitivity for detecting neuroendocrine differentiation in NENs increased from 96.6% (Syn and CgA) to 97.2% (Syn, CgA, and INSM1). Whether INSM1 could be a complementary marker for LCNEC need more research. Thyroid transcription factor 1 (TTF-1) was positively expressed in 40−50% of LCNEC cases (10). Achaete-scute homolog 1 (ASCL1) could also be detected in most LCNECs (17), suggesting TTF1 and ASCL1 were the potential diagnostic markers for LCNEC, exhibit promising prospects in the field of research. Ye and colleagues conducted IHC staining on adenocarcinoma, squamous cell carcinoma, and various types of pulmonary NENs, including lung typical carcinoid (TC), SCLC, and LCNEC, revealing that the expression of human achaete-scute homolog 1 (hASH1), a regulator of neuroendocrine cells growth, was higher in LCNEC (72.7%) and SCLC (79.2%). It was considered that hASH1 could serve as a potential diagnostic marker for HGENC (18). ISL LIM homobox 1 (ISL1), secretagogin (SECG) and syntaxin were proposed for identifying NENs, while the accuracy needs to be fully validated in large case series (19).

The exploration of diagnostic markers for LCNEC and SCLC (20–22) or NSCLC (23) is also crucial. Bari et al. (24) performed RNA sequencing and IHC staining, revealing significant differential expression of caudal type homeobox 2 (CDX2), Villin 1 (VIL1), and brain-specific angiogenesis inhibitor 3 (BAI3) between the cohorts of LCNEC (*n* = 71) and SCLC (*n* = 76). CDX2 and VIL1 in combination had sensitivity and specificity of both 81% for LCNEC while BAI3 showed 89% sensitivity and 75% specificity for SCLC. Therefore, CDX2, VIL1, and BAI3 have the potential to distinguish LCNEC from SCLC. Fan et al. (25) reported that CK7 exhibited negative or weakly positive expression in 88.9% of SCLC cases, whereas 90.8% of LCNEC cases demonstrated partial or complete membrane reported that CK7 exhibited negative or weakly positive expression in 88.9% of SCLC, whereas partial or complete membrane-like positivity with strong intensity in 90.8% of LCNEC, and also had a clear staining pattern in the mixed carcinoma of SCLC and LCNEC. Therefore, CK7 is considered to have a high differential value in SCLC and LCNEC. Stathmin1 (STMN1) mediates cell division and proliferation by regulating microtubule dynamics (26). The expression of STMN1 in HGNEC tissues was found to be significantly higher than NSCLC and showed a high-precision AUC (AUC: 0.984, cutoff: 8.667, sensitivity 92.3%, specificity 95.1%), which could be useful for the differential diagnosis of NSCLC and HGNEC, especially LCNEC (27). The future research should focus on further exploring the gene and protein characteristics of LCNEC, as well as conducting rigorous clinical validation to identify more sensitive and specific diagnostic markers.

## 3 Molecular characterization and subtypes

### 3.1 Molecular characteristic

The genetic alteration of LCNEC has been partially elucidated which is crucial for the therapeutic strategies of LCNEC. Results from genetic sequencing revealed a high prevalence of TP53 (46.4−92%) and RB1 (26−42%) gene mutations (23, 28, 29). Bi-allelic alterations in both genes were observed in 40% of LCNEC. STK11(30%), KEAP1(22%), and PTEN (7%) gene mutations were also common (30). Combined with loss-of-heterozygosity (LOH), bi-allelic alterations of serine/threonine kinase 11 (STK11) and kelch-like ECH associated protein 1 (KEAP1) were identified in 37% of the cases. The somatic alterations of RB1 and STK11/KEAP1 were detected in 82% of the cases (*n* = 49) and occurred in a mutually exclusive pattern (23). Alterations in specific functional genes, such as CREBBP, EP300, NOTCH, MEN1, and ARID1A had also been reported in LCNEC (28, 29, 31–34). Other specific mutations including SMARCA2 (11%), neurotrophic receptor tyrosine kinase 2 (NTRK2) and 3 (NTRK3) (19%) genes (33). FGFR2 was identified as a distinct gene mutation site in LCNEC compared to other NENs (32). Furthermore, pure LCNEC displayed characteristic changes resembling those observed in typical adenocarcinoma, such as EGFR mutation (35, 36), ALK rearrangement (37), RAS pathway mutation, and BRAF mutation (29). This distinguished itself from SCLC as the SCLC lacks these driver gene mutations (38–40). Karlsson et al. (30) used massive parallel sequencing for investigating mutations in 26 cancer-related genes and gene fusions in a cohort of 41 cases of large cell carcinoma (LCC) and 32 cases of LCNEC. The results unveiled TP53 (83%), KRAS (22%), and MET (12%) as the most frequently mutated genes in LCC, while TP53 (88%), STK11(16%), and PTEN (13%) exhibited higher prevalence in LCNEC, no ALK, RET or ROS1 fusions were detected in either LCC or LCNEC.

Gene amplification also occurs frequently in LCNEC with high rates of SRY-box 2 (SOX2) (11%), cyclin E1 (CCNE1) (9%), and MYCN (2%) genes (41). In addition, 4% of LCNEC patients had an increased copy number of ERBB2 and SETBP1 (28, 42). As a downstream target of ASCL1, DLL3 was involved in the regulation of neuroendocrine differentiation (43). Hermans et al. (17) found that expression of DLL3 was positive in 74% (70/94) of LCNEC and higher in 89% (8/9) of TP53 wild-type LCNEC than in 50% (29/58) of mutant-type (*P* = 0.035), indicating that DLL3 played a potential role in identifying molecular subtypes of LCNEC. Klotho served as a potential tumor suppressor in a variety of tumors, inhibited cell proliferation and induced tumor apoptosis (44). It was found that the overall survival (OS) of LCNEC patients with Klotho positive expression was significantly longer than that of Klotho negative (*P* = 0.015, HR = 0.37, 95%CI: 0.17−0.86) (45).

Since the difficulty in obtaining and inadequate tissue of LCNEC, cell-free DNA (cfDNA) analysis has demonstrated great potential in genomic profiling. Zhuo et al. (46) applied next-generation sequence (NGS) to detect cfDNA and the genomic analysis of patients with LCNEC, and showed that the mutation landscape of cfDNA were 90% consistent with tumor DNA, suggesting cfDNA sequencing may be a reliable alternative for genome profiling of LCNEC. In addition, cfDNA sequencing is non-invasive and “real-time,” which makes it an ideal tool for monitoring response and investigating genomic evolution during treatment. Designing the prospective clinical trials to guide treatment based on cfDNA sequencing can help accelerate its clinical application.

### 3.2 Molecular subtypes

As a highly heterogeneous tumor, the categorization of LCNEC based on distinct genomic characteristics holds potential for guiding treatment optimization. Various studies have suggested classification methods for genotyping, thereby determining the response of subtypes to chemotherapy regimens and diverse prognosis (46).

The LCNEC tissues were subjected to NGS sequencing by Rekhtman et al. (33), resulting in the classification of LCNEC into three subtypes. Among the main molecular subtypes, one exhibited SCLC-like features characterized by *TP53/RB1* co-mutation or deletion, accompanied by amplification of *MYCL*, *SOX2*, and *FGFR1* as well as *PTEN* mutation/deletion, and shared several clinicopathological characteristics with traditional SCLC, including higher proliferation activity and shorter relapse-free survival (RFS). The other subtype displayed NSCLC-like features with intact *TP53/RB1* function, *NOTCH* mutations, and genes commonly found in NSCLC such as *STK11/KRAS/TTF1* mutations resembling adenocarcinoma or *KEAP1* mutations resembling squamous cell carcinoma, or *SOX2/FGFR1* amplification. There was evidence suggesting that SCLC-like subtype of LCNEC demonstrated greater sensitivity to platinum-based chemotherapy compared to other subtypes based on a small subgroup analysis. Furthermore, a third subtype was identified with a lower mutation burden, and shared genetic characteristics similar to TC along with *MEN1* gene mutation. Subsequently, George et al. (23) concluded that LCNEC constituted own category within the expression profile domain which differed from NSCLC and TC but bears resemblance to SCLC instead. LCNEC was divided into two independent subtypes, type I was similar to classic SCLC subtype, characterized by alterations in *STK11/KEAP1*, neuroendocrine phenotype with high expression of ASCL1 and DLL3, down-regulating NOTCH pathway, while type II was characterized by changes in *TP53/RB1* displaying predominantly non-neuroendocrine phenotype, with low expression of CgA and Syn, low expression of ASLC1 and DLL3 alongside activation of NOTCH pathway. Zhuo et al. (46) stratified 63 LCNEC patients into SCLC-like and NSCLC-like subtypes based on genomic features derived from tumor DNA and/or cfDNA, and administered chemotherapy regimens of SCLC and NSCLC accordingly. The findings demonstrated varying sensitivities of patients to different chemotherapy regimens, indicating the potential guiding significance of genotyping in treatment selection for improved therapeutic outcomes. However, there is few studies on treatment strategies based on classification and effectiveness, more effective classification methods and corresponding treatment regimens are warranted.

## 4 Treatment

Currently, there is a lack of prospective clinical trial data to support the optimal treatment approach for LCNEC. Approximately 25% of LCNEC patients are early-stage and primarily undergo surgical treatment, while 20% are locally advanced, and 45−50% are advanced patients requiring multidisciplinary treatment. Local radiotherapy may be considered for post-operative or inoperable locally advanced patients, however, the effectiveness of radiotherapy in LCNEC remains uncertain. Due to most of patients being diagnosed at intermediate or late stages, systemic treatment should be combined to enhance therapeutic outcomes. At present, there is a gradual increase in LCNEC patients benefiting from immunotherapy and targeted therapies, and further confirmation is still warranted.

### 4.1 Surgery and radiotherapy

The optimal therapeutic approach for patients with early LCNEC is surgical intervention (47). A previous study data from the SEER database including 1619 patients with stage I-III LCNEC to demonstrate that mOS in surgical patients (41.0°months, 95% CI, 34.9−47.1°months) was significantly longer than in non-surgical patients (12.0°months, 95% CI, 10.3−13.7°months) (*P* < 0.05) (48).

For patients with locally advanced or inoperable LCNEC, local radiotherapy may be considered, whereas the efficacy remains uncertain. The above study retrospectively analyzed patients with stage I-III LCNEC and found that lobectomy had the most favorable impact on OS and lung cancer-specific survival (LCSS) for stage I-II LCNEC. For patients with stage III LCNEC, radiotherapy was significantly associated with an improvement in survival time (48). However, in patients undergoing surgery, post-operative radiotherapy appeared to shorten survival time, suggesting limited additional benefit in improving prognosis.

### 4.2 Chemotherapy

Chemotherapy combined with surgical treatment has significantly enhanced the survival rate of LCNEC. Several studies have investigated post-operative adjuvant regimens. In a retrospective study, Kujtan et al. (49) analyzed 1232 LCNEC patients, among whom 957 (77.7%) underwent surgical resection alone while 275 (22.3%) received EC regimens combined with surgery. Patients who received chemotherapy had a significantly improved 5-year OS rate compared to those who underwent surgery alone (64.5 vs. 48.4%, *P* < 0.001). Multivariate Cox model confirmed that chemotherapy was beneficial for the survival of resected patients with stage I (HR = 0.54, 95%CI: 0.43−0.68, *P* < 0.0001). The efficacy of post-operative adjuvant therapy with chemotherapy regimens for NSCLC/SCLC in patients with LCNEC was retrospectively analyzed by Rossi et al. (8). The results showed a significant prolongation in mOS of LCNEC patients with SCLC regimens (EP) compared to non-treatment (42 vs. 11°months, *P* < 0.001), suggesting that EP should be preferred as the usual regimen for LCNEC. Because of the similarity of genetic and neuroendocrine features between LCNEC and SCLC, the design of clinical trials for LCNEC have mostly favored SCLC. A prospective study demonstrated the addition of adjuvant chemotherapy EP regimen was superior to surgery alone, resulting in a significant improvement in 2-year disease-free survival (DFS) rates (86.7 vs. 47.8%, *P* = 0.0133) and OS rates (88.9 vs. 65.2%, *P* = 0.0252) (50). The phase III clinical trial (51) enrolled 221 patients with post-operative HGNEC, including 30 cases of LCNEC and randomly assigned into EP (*n* = 111) and irinotecan plus cisplatin (IP, *n* = 110). The median follow-up time was 24.1°months, and the 3-year RFS rates were 65.4% for EP and 69.0% for IP (*P* = 0.619), hinting IP was not superior to EP in improving RFS in completely resected HGNEC patients, and EP remained the standard adjuvant therapy option for patients with LCNEC.

The choice of chemotherapy regimens remains controversial for advanced patients with LCNEC. The conventional chemotherapy regimens used for NSCLC and SCLC are also employed in LCNEC, however, the overall efficacy of LCNEC is inferior to that in NSCLC and SCLC (3). The 2023 national comprehensive cancer network (NCCN) guidelines recommend multiple chemotherapy regimens for the systemic treatment of locally unresectable or metastatic pulmonary NEC, including LCNEC. However, these regimens have limited efficacy as second- or subsequent-line therapies, resulting in unsatisfactory patient survival rates and a scarcity of immunotherapy options. According to the 2015 American Society of Clinical Oncology (ASCO) guidelines for systematic treatment of stage IV NSCLC, etoposide plus cisplatin/carboplatin or alternative therapy of non-squamous cell carcinoma was recommended as a first-line approach for patients with advanced LCNEC, nevertheless, the efficacy of this treatment remained uncertain (52). Sun et al. (53) retrospectively analyzed 45 patients with advanced LCNEC, who were divided into SCLC (*n* = 11) and NSCLC regimens (*n* = 34) groups according to first-line chemotherapy regimens. The results showed that the median progression-free-survival (mPFS) of SCLC and NSCLC groups were 6.1 and 4.9°months, respectively, (*P* = 0.41). There was also a considerable difference in the type and efficacy of chemotherapeutic regimens between the two groups, salvage regimens containing irinotecan, platinum, or taxanes had high objective responses in the SCLC group, while frequently used agents in the NSCLC group such as pemetrexed, gefitinib, or erlotinib showed no objective response. Derks et al. (54) conducted a retrospective analysis on 128 patients with advanced LCNEC and categorized them into three groups. First-line use of platinum-based combination chemotherapy, including gemcitabine, docetaxel, paclitaxel, or vinorelbine, was classified as “NSCLC-t,” pemetrexed monotherapy as “NSCLC-pt,” and etoposide chemotherapy as “SCLC-t.” The results demonstrated that the mOS of NSCLC-t chemotherapy was 8.5°months (95% CI: 7.0−9.9), which significantly exceeded the 5.9°months (5.0−6.9; HR = 2.51; 95%CI, 1.39−4.52; *P* = 0.002) of NSCLC-pt treatment and 6.7°months (5.0−8.5; HR = 1.66; 95% CI, 1.08−2.56; *P* = 0.020) of SCLC-t chemotherapy, indicating that NSCLC-t exhibited superior efficacy in advanced LCNEC. In a phase II clinical trial conducted by Niho et al. (55), 44 patients with advanced LCNEC were enrolled, and the results demonstrated the efficacy of irinotecan and cisplatin. The response rate (RR) was 54.5% (95% CI: 38.8−69.6%), while the mPFS and mOS were 5.9°months (95% CI: 5.5−6.3) and 15.1°months (95% CI: 11.2−19.), respectively. Zhuo et al. (46) demonstrated that SCLC-like LCNEC patients (*n* = 15, 24%) treated with EP exhibited superior disease control rates (DCR) compared to those treated with pemetrexed-platinum and GEM/TAX-platinum (100 vs. 20%, *P* = 0.007). For NSCLC-like LCNEC patients (*n* = 48, 76%), treatment with EP or pemetrexed-platinum resulted in longer PFS than GEM/TAX-platinum (5.5 vs. 2.5°months, *P* = 0.045), and there was a trend toward prolonged OS as well (19.6 vs. 9.4°months, *P* = 0.07). Currently, the treatment of LCNEC relies on retrospective and small-scale studies, yielding inconsistent findings that may be attributed to the differential molecular characteristics of LCNEC. In *RB1* wild-type LCNEC, patients treated with the NSCLC regimen (GEM/TAX-platinum) demonstrated significantly improved PFS and OS compared to those receiving the EP regimen (34). In a study by Ito et al. (56), YAP1 expression was detected in 30 patients with LCNEC, with 18/30 (60%) showing YAP1 deletion. Additionally, a significant correlation between YAP1 deletion and neuroendocrine markers expression was observed. Survival analysis revealed that YAP1-negative patients exhibited increased chemotherapy sensitivity. EP regimens are more extensively used in LCNEC, however, prospective large-scale clinical studies are still warranted to provide the improved treatment protocols and the significance of molecular characteristics of LCNEC in guiding precision chemotherapy regimens.

### 4.3 Immunotherapy

Immunotherapy had demonstrated comparable therapeutic efficacy on NSCLC and SCLC, while limited studies had been conducted on LCNEC, which is currently under clinical trial investigation. LCNEC cells exhibit a high expression level of PD-L1, along with a median tumor mutational burden (TMB) of 9.9 muts/Mb, suggesting the potential effectiveness of immunotherapy in patients with LCNEC. Dudnik et al. (57) reported that the mOS for patients (*n* = 41) with LCNEC who received immune checkpoint inhibitors (ICIs) from diagnosis or initiation of treatment was 12.4 or 11.0°months, respectively. In contrast, the mOS for non-ICI regimens among 84 patients was 6.0°months. The retrospective study conducted by Komiya et al. (58) revealed that immunotherapy resulted in a significant improvement in OS among patients with stage IV LCNEC (*n* = 37), compared to those who did not receive immunotherapy (*n* = 624). The 12-month survival rate was 34.0% versus 24.1%, while the 18-month survival rate stood at 29.1% versus 15.0% in the patients treated with or without immunotherapy. Furthermore, Oda et al. (59) reported a case of obtaining complete response (CR) for up to 4°years after treatment with pembrolizumab. According to the report of Zhang et al. (60), pembrolizumab plus endostar was administered as first-line therapy for a patient diagnosed with LCNEC, the PFS was 2°years. Sherman et al. (61) conducted a clinical trial in which a total of 37 patients with advanced LCNEC were enrolled, patients (*n* = 21) who received anti-PD-1/PD-L1 immunotherapy demonstrated an ORR of 33%, a complete response rate (CRR) of 11%, a mPFS of 4.2°months (95% CI, 2.4−8.1), and a mOS of approximately 11.8°months (95% CI, 3.7-NR). These findings were consistent with the previous report on NSCLC and LCNEC, indicating similar effectiveness between LCNEC and NSCLC in the context of immunotherapy. The prospective multicenter phase II clinical trial by Patel et al. (62) enrolled 32 patients diagnosed with neuroendocrine tumors to assess the efficacy of the treatment with anti-PD-1 plus anti-CTLA-4 antibodies. Among these cases, 18 (56%) patients were classified as HGNEC. The results showed the ORR was 25%, the 6-month PFS rate was 31% (95% CI, 19−52%), and the mOS was 11°months of the patients with HGNEC, and there was no significant increase in adverse reactions compared with other patients. However, the current study had a short follow-up time and did not evaluate long-term toxic effects such as pulmonary fibrosis and esophageal stenosis or fistula formation.

Nowadays, immunotherapy plus chemoradiotherapy remains the main research direction of LCNEC. Levra et al. (63) conducted a retrospective analysis of 10 patients with stage IIIB-IV LCNEC who received nivolumab or pembrolizumab after first-line treatment with platinum, and revealed an ORR of 60% and a mPFS of 14°months. Song et al. (64) retrospectively analyzed 10 cases of LCNEC treated with pembrolizumab plus chemotherapy as the first-line treatment, the mPFS was 5.5°months (95%CI, 2.3−8.7), mOS was 13.0°months (95%CI, 11.0−15.0), ORR and DCR were 70 and 90%, respectively. In 8 patients who treated with the pembrolizumab with or without chemotherapy as second-line or subsequent treatment, the mPFS was 3.8°months (95%CI, 0.0−7.6) and mOS was not reached, suggesting that pembrolizumab plus chemotherapy may be an attractive first-line treatment strategy for improving survival outcomes in patients with advanced LCNEC. Meanwhile, the use of pembrolizumab plus chemotherapy as first-line therapy was found to be associated with improved PFS in patients with PD-L1 expression ≥50% (*P* = 0.024). Chauhan et al. (65) reported three LCNEC patients who received nivolumab after progression of platinum-based chemotherapy, all of whom achieved a stable and durable response. Mauclet et al. (66) also reported a case of locally advanced LCNEC achieving complete tumor response after palliative thoracic radiotherapy and nivolumab therapy. Immunotherapy holds promise as a treatment for LCNEC and has the potential to overcome current therapeutic limitations.

Although the above findings indicated that LCNEC patients respond well to immunotherapy, it existed divergent outcomes. The phase I study conducted by Kim et al. (67) included 9 cases of pulmonary HGNEC, the nivolumab plus lutetium-labeled somatostatin analog (lutathera) was administered. Despite exhibiting favorable tolerability, the anti-tumor activity of the drugs demonstrated limitations, necessitating the expansion of sample size. Han et al. (68) reported a case of LCNEC patient treated with post-operative chemotherapy followed by bevacizumab, paclitaxel, and nivolumab. Despite the positive PD-L1 expression, the patient exhibited rapid disease progression. Delayed initiation of immunotherapy may limit efficacy in such cases.

Immunotherapy may overcome existing therapeutic limitations of LCNEC, exploring efficacy and prognostic markers that can identify beneficiary populations or responses and are crucial for achieving precise treatment. For instance, inflammation and immune biomarkers such as the neutrophil-lymphocyte ratio (NLR) and platelet-lymphocyte ratio (PLR) in peripheral blood may be potential biomarkers for predicting the efficacy of immunotherapy in LCNEC (69). Okui et al. (70) demonstrated that preoperative NLR was an independent prognostic factor for OS in LCNEC (HR = 2.46, 95%CI, 1.508−4.011, *P* < 0.001). Christopoulos et al. (71) found that mild lymphocyte depletion commonly occurred in LCNEC, while patients who exhibited significant alterations in T-cell repertoire (TCR) subsets and higher lymphocyte counts prior to treatment demonstrated improved outcomes and longer survival time (441 vs. 157°days, *P* = 0.019). Thus, TCR could be exploited for prognostic, predictive and therapeutic purposes. Chen et al. (72) reported that *KEAP1* mutation was a potential therapeutic biomarker in patients undergoing immunotherapy. In other types of lung cancer, immunotherapy efficacy-related markers include PD-L1, TMB, MSI/dMMR, *POLE/POLD1* mutations, etc (73). Nevertheless, single and generic predictive markers may not be able to predict the therapeutic efficacy accurately, the specificity of biomarkers could vary depending on tumor type and different ICIs, so relevant predictive markers of efficacy in LCNEC remain to be explored.

### 4.4 Targeted therapy

Large cell neuroendocrine carcinoma (LCNEC) contains potentially targetable gene alterations, including *EGFR* mutations ( ± 2%), *BRAF* mutations ( ± 1%) and *FGFR1* amplification ( ± 3%), and these potential therapeutic targets are more frequent in *RB1* wild type (84%) than in *RB1* mutant type (50%) tumors (23). Gefitinib and icotinib, the first-generation EGFR tyrosine kinase inhibitors (TKIs), were effective in the treatment of LCNEC with *EGFR* exon 19 deletion, and the response lasted for 6 and 8°months, respectively, (35, 74). Ricco et al. (75) demonstrated that the LCNEC with *BRAF* V600E (G469R) mutation responded to BRAF/mitogen-activated protein kinase inhibitors. *ALK* rearrangement rarely occurs in LCNEC (37, 76). LCNEC patients with *ALK* rearrangement showed regression in both the primary site and multiple metastatic sites after receiving ALK-TKI treatment (76). Therefore, the second or third generation ALK TKIs, such as ceritinib, brigatinib, and loratinib, may be the options of LCNEC patients with *ALK* rearrangement and secondary central nervous system involvement.

Alteration of genes associated with *PI3K-AKT-mTOR* pathway was common in LCNEC, and patients with these mutations may respond to targeted therapy. In a clinical trial of phase II (77), 49 patients with metastatic LCNEC were enrolled in the first-line treatment with everolimus plus paclitaxel and carboplatin. The outcomes showed that the ORR was 45% (95%CI, 31−60%), the DCR was 74% (95%CI, 59−85%), the mPFS was 4.4°months (CI, 3−6), the mOS was 9.9°months (CI, 6.9−11.7), and grade 3/4 toxicity occurred in 51% of patients, suggesting that everolimus plus carboplatin and paclitaxel was considered to be the effective and well tolerated first-line treatment for metastatic LCNEC. Rossi et al. (8) conducted a review on 83 LCNEC patients, the histological analysis of LCNEC indicated a strong expression of receptor tyrosine kinases (RTKs), including KIT (62.7%), PDGFRα (60.2%), PDGFRβ (81.9%), and Met (47%), suggesting RTKs could serve as potential therapeutic targets, while no mutation was detected in the exons encoding for the relevant juxta membrane domains. Delta-like ligand 3 (DLL3) is an inhibitory Notch ligand with extremely low expression in normal tissues but high expression in LCNEC and SCLC (78). Saunders et al. (78) demonstrated that the ADC drug SC16LD6.5 (rovalpituzumab tesirine, Rova-T) effectively targeted and eradicated DLL3-expressing initiating cells (TICs) in SCLC and LCNEC patient-derived xenograft (PDX) tumors and was a promising first-in-class ADC for the treatment of HGNEC (79). Although the clinical trial and several other studies investigating ADC agents targeting DLL3 in lung cancer did not meet anticipated outcomes (80, 81) the ongoing clinical investigations on drugs targeting DLL3 are numerous. Subsequently, study on phase I by Morgensztern et al. (82) applied SC-002, a DLL-3 targeted drug, to treat SCLC and LCNEC. The results revealed that SC-002 had systemic toxicity and limited efficacy, similar to the Rova-T ADC, indicating that this drug is unlikely to achieve the requisite level of benefit in this difficult-to-treat patient population. At present, the global pipeline is still working on three projects that activate T cells through CD3 and specifically target cancer cells with high expression of DLL3, which have shown good anti-tumor efficacy in preclinical and clinical stages. The bispecific antibody may be promising in the treatment of LCNEC.

The investigation of immune and targeted therapy in the management of LCNEC has emerged as a prominent area of research. Table 3 presents an overview of ongoing clinical trials focusing on advanced and metastatic LCNEC.

**TABLE 3:** Ongoing clinical trials in Large cell neuroendocrine cancer (LCNEC).

NCT	Phase	*N*	Tumors	Setting	Experimental arm	Primary endpoint	Status	Study completion
Eudract 2020-005942-41 (DUPLE)	II	49	LCNEC	1st-line	Durvalumab+carboplatin°+ °etoposide × 4 → durvalumab	1-year OS rate	Recruiting	2026-07
NCT05126433	II	60	Poorly differentiated Neuroendocrine tumors, including LCNEC	Progressed on platinum-based regimen (irrespective of number of prior lines)	Lurbinectidin	ORR	Recruiting	2024-06
NCT03591731	II	185	Poorly differentiated neuroendocrine tumors, including LCNEC	Progressed after one or two lines of treatment, including at least one line of platin-based chemotherapy	Arm A: Nivolumab Arm B: Nivolumab°+°Ipilimumab	ORR	Active, not recruiting	2023-09
NCT03728361	II	55	Cohort 1: SCLC; Cohort 2: Metastatic NEC of any grade/primary site (including LCNEC)	Cohort 1: progressed or recurred after platinum-based chemotherapy with immunotherapy; Cohort 2: Any line	Nivolumab°+°Temozolomide	ORR	Active, not recruiting	2023-12
NCT05262985	II	22	LCNEC	Single Arm: patients with advanced treatment naive LCNEC	Durvalumab°+°Cisplatin/ Carboplatin+Etoposide	PFS	Recruiting	2025-01
NCT05470595	II	67	LCNEC	Single Arm: Patients with locally advanced or metastatic LCNEC	Atezolizumab/Platinum/Etoposide	OS	Recruiting	2029-01
NCT05546268	I	133	HGNEC (including LCNEC)	High-grade neuroendocrine cancer of any primary sites	Oral MRT-2359 (MYC-Driven)	DLT/MTD/RP2D	Recruiting	2027-11
NCT05652686	I	58	Advanced Refractory Cancers (including LCNEC)	Histologically or cytologically confirmed unresectable advanced or metastatic LCNEC	PT217 (DLL-3/CD47)	DLT/MTD/RP2D	Not yet recruiting	2025-06
NCT05680922	I	41	LCNEC	DLL3-targeted chimeric antigen receptor T-cells in subjects with LCNEC	LB2102 (DLL3-Directed CART)	RDE	Not yet recruiting	2028-03
NCT04429087	I	193	NEC (including LCNEC)	Single Arm: Patients with locally advanced or metastatic LCNEC	BI 764532 (DLL3/CD3 bispecific)	MTD	Recruiting	2024-09

LCNEC, large cell neuroendocrine cancer; NEC, neuroendocrine carcinoma; HGNEC, high-grade neuroendocrine carcinoma; ORR, objective response rate; PFS, progression-free survival; DLT, dose-limiting toxicity. MTD, maximum tolerated dose; RP2D, recommended Phase II dose; RDE, recommended dose for expansion; DLL3, delta-like ligand 3.

## 5 Conclusions and perspectives

Large cell neuroendocrine carcinoma (LCNEC) is a rare neuroendocrine carcinoma with high heterogeneity. According to the gene status of *TP53* and *RB1*, researchers classified LCNEC to SCLC-like type and NSCLC-like type, and initially explored the sensitivity of different subtypes to chemotherapy regimen. However, the results still need more clinical data to confirm. Since there is no standard treatment currently, clinical trials of immunotherapy and targeted therapy in LCNEC had exhibited promising outcomes, instilling optimism for enhancing the survival rates of patients.

The focus of future research should be directed toward elucidating the molecular mechanisms and characteristics of LCNEC, with the aim of identifying more effective molecular targets to overcome therapeutic challenges. It is imperative to discover novel treatment modalities by exploring combination strategies involving ICIs, new immunotherapeutic agents, and other innovative approaches. Additionally, researchers should evaluate the efficacy of ICIs in larger patient cohorts and establish correlations between genomic features and treatment responses, thereby facilitating informed decision-making. Furthermore, investigating molecular biomarkers associated with immunotherapy and profiling the molecular characteristics of patients who derive survival benefits will greatly enhance therapeutic outcomes through personalized treatment strategies for LCNEC.

## Author contributions

SZ: Writing – original draft. XW: Writing – original draft. HL: Writing – original draft. PZ: Writing – review & editing. JL: Writing – original draft. LZ: Writing – review & editing. YC: Writing – review & editing.
